# Chitosan oligosaccharide improves ovarian granulosa cells inflammation and oxidative stress in patients with polycystic ovary syndrome

**DOI:** 10.3389/fimmu.2023.1086232

**Published:** 2023-03-01

**Authors:** Qi Xie, Wenli Hong, Yuan Li, Shuyi Ling, Ziqiong Zhou, Yuqing Dai, Wenbo Wu, Ruoxin Weng, Zhisheng Zhong, Jun Tan, Yuehui Zheng

**Affiliations:** ^1^ Reproductive Health Department, Shenzhen Traditional Chinese Medicine Hospital, The Fourth Clinical Medical College of Guangzhou University of Traditional Chinese Medicine, Shenzhen, China; ^2^ Jiangxi Provincial Key Laboratory of Reproductive Physiology and Pathology, Nanchang University, Nanchang, China; ^3^ Reproductive Medicine Center, Xinyu Maternal and Child Health Care Hospital, Xinyu, China; ^4^ Shenzhen University Health Science Center, Shenzhen University, Shenzhen, China; ^5^ Reproductive Medicine Center, Jiangxi Maternal and Child Health Hospital, Nanchang, China

**Keywords:** polycystic ovary syndrome, granulosa cells, chitosan oligosaccharide, inflammation, oxidative stress

## Abstract

**Introduction:**

Polycystic Ovary Syndrome (PCOS) is the most common reproductive endocrine disorder among women of reproductive age, which is one of the main causes of anovulatory infertility. Even though the rapidly developed assisted reproductive technology (ART) could effectively solve fertility problems, some PCOS patients still have not obtained satisfactory clinical outcomes. The poor quality of oocytes caused by the abnormal follicular development of PCOS may directly contribute to the failure of ART treatment. Ovarian granulosa cells (GCs) are the most closely related cells to oocytes, and changes in their functional status have a direct impact on oocyte formation. Previous studies have shown that changes in the ovarian microenvironment, like oxidative stress and inflammation, may cause PCOS-related aberrant follicular development by impairing the physiological state of the GCs. Therefore, optimizing the ovarian microenvironment is a feasible method for enhancing the development potential of PCOS oocytes.

**Methods:**

In this study, we first detected the expression of inflammatory-related factors (TGF-β1, IL-10, TNFα, IL-6) and oxidative stress-related factors (HIF-1α and VEGFA), as well as the proliferation ability and apoptosis level of GCs, which were collected from control patients (non-PCOS) and PCOS patients, respectively. Subsequently, human ovarian granulosa cell line (KGN) cells were used to verify the anti-inflammatory and anti-oxidative stress effects of chitosan oligosaccharide (COS) on GCs, as well as to investigate the optimal culture time and concentration of COS. The optimal culture conditions were then used to culture GCs from PCOS patients and control patients.

**Results:**

The results showed that GCs from PCOS patients exhibited obvious inflammation and oxidative stress and significantly reduced proliferation and increased apoptosis. Furthermore, COS can increase the expression of anti-inflammatory factors (TGF-β1 and IL-10) and decrease the expression of pro-inflammatory factors (TNFα and IL-6), as well as promote the proliferation of GCs. Moreover, we found that COS can reduce the level of reactive oxygen species in GCs under oxidative stress by inhibiting the expression of HIF-1α and VEGFA and by suppressing the apoptosis of GCs induced by oxidative stress.

**Conclusion:**

We find that inflammation and oxidative stress exist in the GCs of PCOS patients, and COS can reduce these factors, thereby improving the function of GCs.

## 1 Introduction

Polycystic ovary syndrome (PCOS) is the most common reproductive endocrine and metabolic disorder leading to infertility in women of childbearing age. It is mainly manifested as hyperandrogenemia (clinical and biochemical), ovarian dysfunction, and polycystic ovary morphology, usually associated with insulin resistance (IR) and obesity. Recently, PCOS was found to be accompanied by oxidative stress and chronic low-grade inflammation. Ovarian dysfunction remains the main feature of the syndrome and the leading cause of anovulatory infertility. The prevalence of the condition is approximately contributing to 10-13% of women of reproductive age and up to 30%-60% of patients with ovulatory dysfunctional infertility (1). In recent years, assisted reproductive technology (ART) has developed rapidly and has become an important way to solve infertility problems in PCOS patients. However, some PCOS patients still do not achieve satisfactory clinical outcomes after ART treatment, which is mainly attributed to the pathological changes of abnormal follicular development (2). However, the related molecular regulatory mechanisms are still not fully understood. Therefore, exploring the molecular mechanisms of abnormal follicle development in PCOS and finding ways to improve follicle quality in PCOS patients are key nodes and potential applications for improving clinical pregnancy outcomes in ART.

During the normal follicular development process, granulosa cells (GCs) are the most important cells, which directly affect the development of the oocyte. Several studies have shown that the GCs dysfunction can result in abnormal follicular development in women with PCOS (3). It is worth noting that aberrant changes in the ovarian microenvironment, such as oxidative stress and inflammation, can impair the physiological state of GCs, which may also be the main cause of subsequent PCOS follicular development disorders (4, 5). Previous studies have found significantly higher levels of inflammation factors and reactive oxygen species (ROS) in PCOS patients’ ovaries when compared with normal women (6, 7), suggesting that the ovarian microenvironment in PCOS patients is in a low-grade chronic inflammation (8). Further studies have confirmed that the increased level of inflammation in follicular fluid can cause GCs dysfunction, which disturb the normal development of oocytes (9). In addition, the increased oxidative stress also induces the apoptosis of GCs (10). A high level of ROS can damage the biological function of GCs, which results in the poor follicle quality (11). All the above studies suggest that inflammation and oxidative stress in GCs are likely to be the crucial reasons causing abnormal follicle development in PCOS and that alleviating the inflammation and oxidative stress in GCs may improve follicular development.

Chitosan oligosaccharide (COS) is mainly derived from crustaceans and also exists in fungi, insects, cell membranes of algae and cell walls of higher plants. Various biological effects of COS have been reported, such as immunomodulatory (12), anti-tumor (13), antibacterial, antifungal (14, 15), antioxidant (16) and anti-inflammatory (17). Several studies have reported that COS can act as a potent free radical scavenger to balance biomolecules in oxidative and antioxidant processes in cellular systems and inhibit intracellular ROS formation (18, 19). Additionally, COS inhibits the inflammatory response of macrophages induced by lipopolysaccharide (LPS) and reduces the expression of pro-inflammatory cytokines TNF-α and IL-6 (13). More importantly, our team found that COS has a protective effect on hydrogen peroxide- (H_2_O_2_-) stimulated oxidative damage in human ovarian granulosa cell line (KGN) (20), and COS can promote the proliferation of ovarian germ stem cells and reshape the ovarian function by improving the ovarian microenvironment and stimulating the secretion of immune related factors (21).

In this study, we firstly compared the clinical data of PCOS patients and normal patients, then collected GCs from both groups to explore the correlation between GCs and inflammation and oxidative stress in PCOS patients. Because of the number of GCs obtained after collection and purification were limited, and they had a limited lifespan *in vitro* under the stimulation of supraphysiological doses of FSH and HCG *in vivo*, therefore, we used KGN cells to investigate the ameliorative effect of COS on inflammation and oxidative stress. KGN is a tumor cell derived from human granulosa cells. It not only has the ability of infinite proliferation, but also has the normal biological function and biological activity contained in normal GCs. KGN as a tool to study granulosa cells has been proved by many studies, and many people use KGN as a tool to study the function and role of GCs (22, 23). Finally, GCs from PCOS patients and control patients were cultured with COS to verify its ameliorating effect on inflammation and oxidative stress in PCOS related GCs.

## 2 Materials and methods

### 2.1 Clinical specimens

Follicular fluid was obtained from patients receiving *in vitro* fertilization or intracytoplasmic sperm injection (ICSI) in Reproductive Medicine Center of Jiangxi Provincial Maternal and Child Health Hospital. After the follicles were fully developed, human chorionic gonadotropin (HCG) was given to promote ovulation and eggs were retrieved 36h later. All samples were collected with written informed consent. Patients with PCOS were diagnosed according to Rotterdam criteria (60 cases). patients with infertility due to fallopian tube factors or male factors were assigned to the normal group (60 cases).

### 2.2 Isolation, extraction, and culture of GCs

The follicular fluid collected after oocyte pick-up (OPU) was centrifuged to discard the supernatant, and the remaining cell precipitates were suspended in the same volume DMEM/F12 culture medium. The cell suspension was transferred to the surface of 50% Percoll (GE) separation solution at a volume ratio of 2:3, and GCs were obtained after centrifugation. Erythrocytes are removed with erythrocyte lysate. Then, the cells are used for culture, protein or RNA extraction, or frozen at -80°C. For culture, the cells were suspended in DMEM/F12 medium containing 10% FBS in a 12-well plate and placed in cell incubator (37°C, 5% CO_2_). After 24 hours, the culture medium was replaced with a fresh medium.

### 2.3 EDU cell proliferation detection

The isolated GCs are collected in EP tubes and the PBS are removed by centrifugation. 100 μL of 50 μM EDU medium are added to each tube and incubated for 2 hours. The mediums are discarded, and the cells are washed once or twice with PBS for 5 mins each time, then are stained after incubation with EDU and incubated with PBS containing 4% paraformaldehyde for 30 mins at room temperature. The cells are washed with PBS for 5 mins and the PBS was discarded. 100 μL of osmolyte was added and incubated for 10 min, the cells are washed once with PBS for 5 mins. 100 μL of 1X Apollo^®^ staining reaction solution are added and incubated for 30 mins at room temperature in a shaker protected from light. DNA staining: add 1X Hoechst33342 reaction solution, incubates for 30 mins at room temperature in a light-proof shaker, discards the staining reaction solution, then washes 1~3 times with PBS, and immediately observe and takes pictures with a fluorescent microscope.

### 2.4 Detection of apoptosis by TUNEL

The naturally dried GCs were incubated with cell fixative for 15 mins at room temperature and then the fixative was removed, incubated with deionized water for 5 mins, and then the deionized water was removed. Permeant was added and incubated for 10 mins at room temperature, then permeant was removed and washed twice with deionized water for 5 mins each. Add 50 μL of 1X TdT Buffer per sample to cover the cells, leave at room temperature for 10 mins and discard, then add 50 μL of TdT enzyme incubation solution and incubate at 37°C for 2 hours. Add 100 μL of 2X SSC and leave at room temperature for 15 mins to terminate the reaction, discard the SSC. Add an appropriate amount of PBS and wash the samples twice for 5 mins each time. Add 100 μL of 1X DAPI reaction solution per sample and incubate for 5 mins at room temperature. Add 100 μL 1X DAPI reaction solution, incubate for 30 mins at room temperature avoiding light in a shaker and discard the staining reaction solution, wash the samples three times with PBS for 5 mins each time, followed immediately by fluorescence microscopy for observation and counting of photographs.

### 2.5 Protein extraction and immunoblotting

RIPA lysate was used to extract GCs proteins, and the extracted proteins were added to the loading buffer and boiled at 95°C for 5min. After that, the extracted proteins were subjected to immunoprotein blotting according to the instructions. The main antibodies used in this study included TGF-β1 (Abcam, AB92486), IL-10 (Abcam, AB34843), TNFα (Abcam, AB6671), IL-6 (Abcam, Ab6672), HIF-1α (Cell Signaling, #3716), VEGFA (Proteintech, 66828-1-LG), and GAPDH (Proteintech, 60004-1-LG), and all secondary antibodies were purchased from Immunoway.

### 2.6 RNA extraction and RT-PCR

The total RNA of GCs was extracted by Trizol method, and the RNA was reverse-transcribed according to the instructions of TaKaRa kit. The cDNA obtained from reverse transcription was amplified to obtain △CT value. The results were calculated and compared using 2-[A-B]-[C-D] (A: average CT value of target genes in the experimental group, B: average CT value of reference genes in the experimental group, C: Average CT value of target genes in the control group, D: average CT value of reference genes in the control group). The primer serial numbers are as follows in **Table 1**.

**Table 1:** The sequences of primers used for q-PCR.

Gene	Forward primer (5’-3’)	Reverse primer (5’-3’)
GAPDH	ACATCGCTCAGACACCATG	TGTAGTTGAGGTCAATGAAGGG
TGF-β1	CTAATGGTGGAAACCCACAACG	TATCGCCAGGAATTGTTGCTG
TNF-α	CCTCTCTCTAATCAGCCCTCTG	GAGGACCTGGGAGTAGATGAG
IL-10	GACTTTAAGGGTTACCTGGGTTG	TCACATGCGCCTTGATGTCTG
IL-6	ACTCACCTCTTCAGAACGAATTG	CCATCTTTGGAAGGTTCAGGTTG
HIF-1α	ATCCATGTGACCATGAGGAAATG	TCGGCTAGTTAGGGTACACTTC
VEGA	AGGGCAGAATCATCACGAAGT	AGGGTCTCGATTGGATGGCA

### 2.7 Cell proliferation assay

After the cells were digested and centrifuged, the cells were repeatedly suspended with an appropriate amount of culture medium, and then the total number of cells was calculated after sample counting under a microscope, and then the cell suspension was diluted to a density of 3×10^4^/mL. The cell suspension was inoculated into 96-well plates (100 μL/well), and the wells were divided into blank group (no cells but only medium), negative control group (same volume of medium without COS), and experimental group (COS concentration of 100, 200, 300 μg/mL medium) according to the experimental design, and five replicate wells were set up for each group. The plates were incubated (37°C, 5% CO_2_) for a period of time according to the experimental design, serum-free medium containing 10% CCK8 was prepared before each assay, and 100μL of the configured medium was added to each well, and the whole process was carried out under light-proof conditions. The plates were incubated for 2 hours under light-proof conditions. After incubation, the OD value of the samples at 450 nm was measured by a microplate reader immediately. The measured OD value was subtracted from the blank group as the final measured value, and the cell proliferation curve was drawn by statistical software.

### 2.8 Flow cytometry

The cultured GCs were digested with trypsin without EDTA, and the cells were centrifuged and washed twice with pre-cooled PBS at 300 g at 2-8 °C for 5 min. Cells were suspended in 400 μL 1× Annexin V binding solution at a concentration of approximately 1×10^6^ cells/mL. 5 μL Annexin V-FITC staining solution was added to the cell suspension, and then the cells were gently mixed and incubated at 2-8 °C under dark conditions for 15 mins. Add 5-10μL PI staining solution, mix it gently, and incubate for 5 mins at 2-8 °C under dark conditions. Then, the solution was detected by flow cytometry immediately.

### 2.9 Reactive oxygen species detection

DCFH-DA was diluted with a serum-free medium at 1:1000 to reach a final concentration of 10μM. Remove the cell culture medium and add an appropriate volume of diluted DCFH-DA, which should be sufficient to cover the cells. A ROSUP control stimulus was added to the positive control group at a ratio of 1:1000. The cells were placed in 37°C culture incubator and incubated for 20 mins. Cells were washed three times with serum-free cell culture medium to adequately remove free DCFH-DA, followed by observation and analysis by fluorescence microscopy.

### 2.10 Statistical analysis

SPSS 26.0 software was used to analyze the data. Independent T test was used to analyze the clinical data conforming to the normal distribution, and non-parametric test was used to analyze the clinical data conforming to the normal distribution (25% and 75% percentile were used to represent the indicators not conforming to the normal distribution). P < 0.05 was statistically significant.

All experiments were repeated at least three times for each group. Data were statistically analyzed by GraphPad Prism6.0 software, and analysis method was ANOVA and T test. P < 0.05, P < 0.01 and P < 0.001 were statistically significant.

## 3 Results

### 3.1 Clinical information analysis of PCOS patients

The clinical characteristics of patients were shown in **Table 2**. BMI, age, AMH, basal LH, basal P, basal T, number of antral follicles in left and right ovary, total number of harvested eggs, MIIcleavage rate and high-quality embryo rate were significantly different between the control group and PCOS group (*P <*0.05). The MIIcleavage rate and excellent embryo rate of MII in control group were significantly higher than those in PCOS group. There were no significant differences in basal FSH and basal E_2_ between the control and PCOS groups (*P* > 0.05).

**Table 2:** Clinical data of control group and PCOS group treated with ART.

	Control group	PCOS group	Z/t	p
Number of cases	60	60		
BMI	22.29 ± 3.24	23.64 ± 3.49	-2.303	0.023
FSH(IU/L)	4.96 ± 1.23	4.82 ± 1.40	0.581	0.562
Age	(28,33)	(24,30.25)	-4.216	0.000
AMH	(2.2,3.67)	(4.98,12.98)	-6.719	0.000
LH(mIU/ml)	(2.285,4.645)	(3.93,11.235)	-5.063	0.000
E2(pg/ml)	(28.5,54.75)	(31.075,46.0)	-0.588	0.557
P(ng/dl)	(0.2,0.3)	(0.1,0.3)	-2.672	0.008
T(ng/dl)	(23.415,34.97)	(33.43,58.15)	-5.374	0.000
Sinus follicles(L)	(6,10)	(10,12)	-6.570	0.000
Sinus follicles(R)	(6,10)	(10,12)	-6.566	0.000
Number of oocytes retrieved	(10,17)	(12,23.25)	-3.422	0.001
Cleavage rate	(0.721,1.0)	(0.713,0.780)	-2.418	0.016
Normal fertilization rate	(0.667,0.90)	(0.634,0.877)	-1.136	0.256
Rate of good quality embryo at day 3	(0.143,0.369)	(0,0.280)	-2.983	0.003

Note: Indicators that do not meet the normal distribution are expressed using the 25% and 75 percentiles.

### 3.2 Expression of factors associated with inflammation and oxidative stress in GCs from PCOS and control group

**Figure 1:**
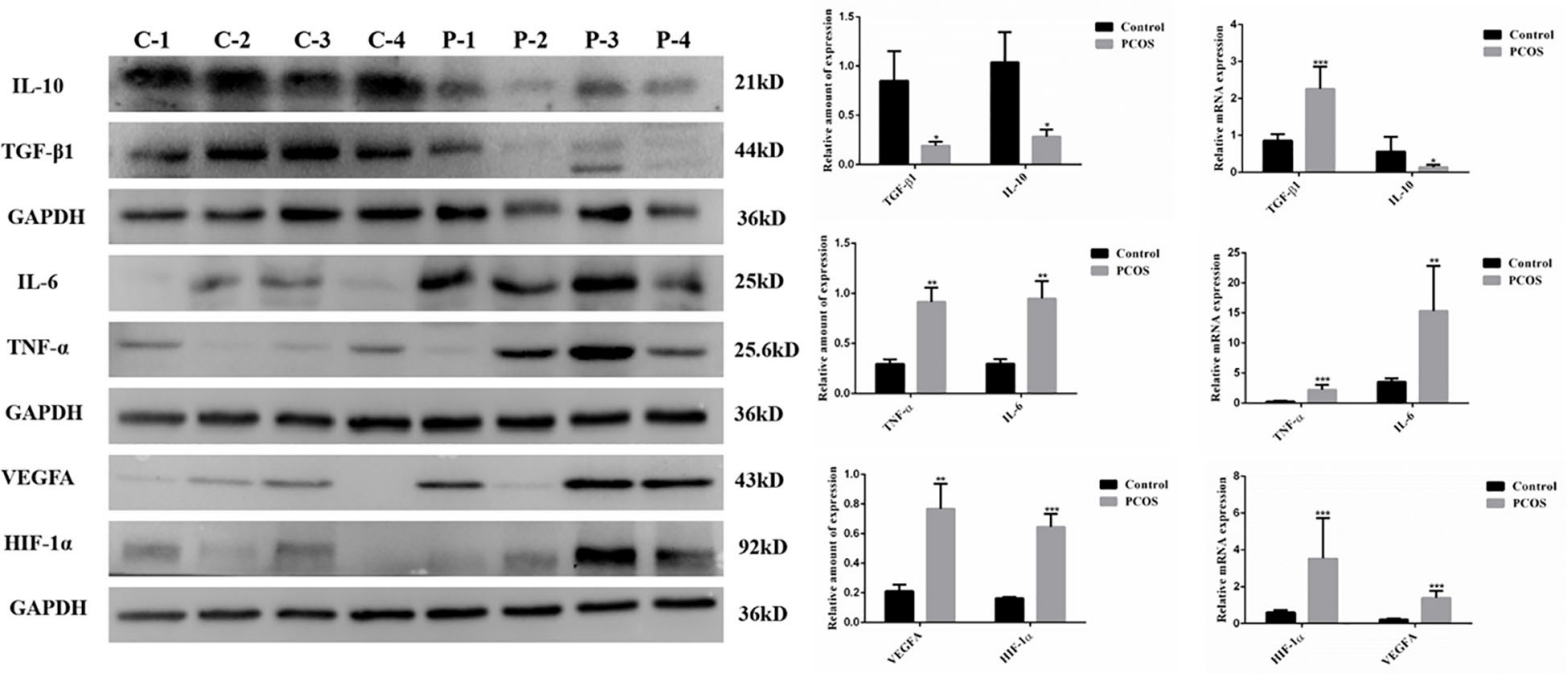
Expression of factors associated with inflammation and oxidative stress in GCs from PCOS (P) and control (C) group. Compared to the control group, the expression of IL10 and TGF-β1, which were related to anti-inflammatory, was significantly decreased in GCs from PCOS group (n=25 to 50 in each group). Meanwhile, the expression of pro-inflammatory cytokines (TNFα and IL-6) and oxidative stress-related factors (HIF-1α and VEGFA) was remarkably increased in GCs from PCOS group when compared with control group (n=25 to 46 in each group). (*P<0.05, **P<0.01, ***P<0.001).

We found that the expression of anti-inflammatory factors TGF-β1 and IL-10 in GCs of PCOS patients was significantly lower than those in control patients. In contrast, PCOS patients showed a higher expression of pro-inflammatory factors TNF-α and IL-6 mRNA and protein when compared to control patients. Furthermore, the expressions of oxidative stress-related factors HIF-1α and VEGFA were also found to be significantly higher in GCs of PCOS patients than those in control patients, indicating that PCOS related GCs were accompanied with inflammation and oxidative stress (**Figure 1**).

### 3.3 Proliferation and apoptosis of GCs from PCOS and control group

**Figure 2:**
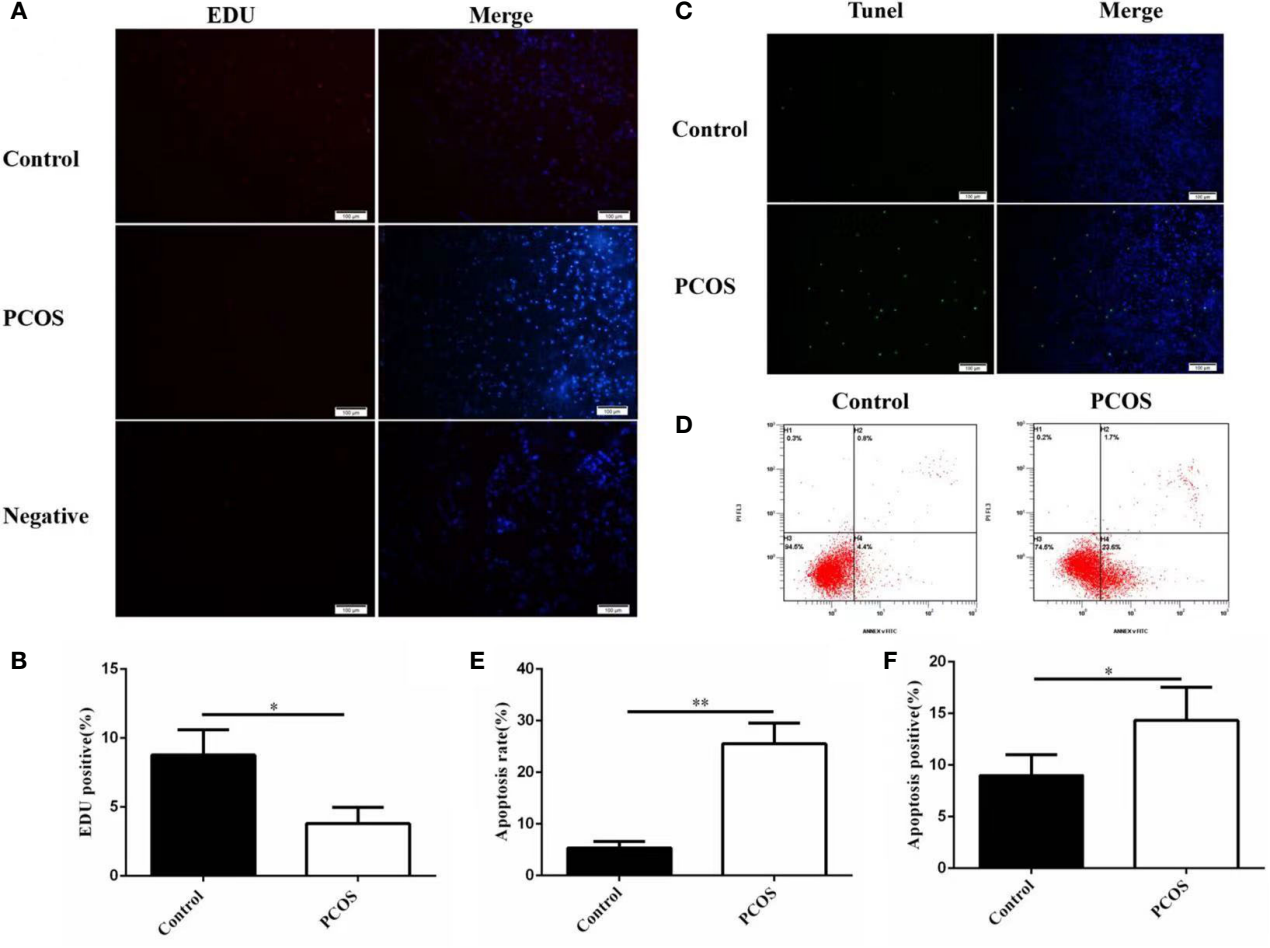
Proliferation and apoptosis of GCs from PCOS and control group. The proliferation ability of granulosa cells in PCOS group was significantly lower than that in control group **(A, B)**, n=5 in each group. The apoptosis level of granulosa cells in PCOS group was significantly higher than that in control group **(C–F)**, n=5 in each group. *P<0.05, **P<0.01 *vs* Control group.

We investigated the proliferation and apoptosis of GCs isolated from two groups. As shown in **Figure 2**, GCs in PCOS group represented lower proliferation(A-B) and higher apoptosis levels compared to control group(C-F), revealing that the growth ability of GCs was remarkably lower in PCOS group.

### 3.4 COS improves inflammatory response and oxidative stress in KGN cells

**Figure 3:**
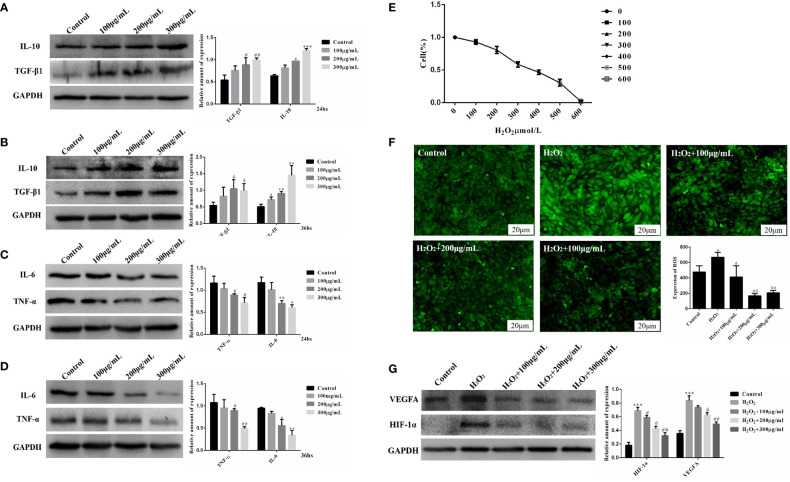
COS improves inflammatory and oxidative stress in KGN cells. COS promotes the expression of anti-inflammatory factors TGF-β1 and IL-10 protein in KGN cells in a dose and time-effect relationship. (Protein expression of TGF- β1 and IL-10 after COS culture for 24 hours **(A)**; Protein expression of TGF- β1 and IL-10 after COS culture for 36 hours **(B)**. COS inhibits the expression of pro-inflammatory factors TNF-α and IL-6 in KGN cells in a dose and time-dependent relationship. (Protein expression of TNF-α and IL-6 after COS culture for 24 hours **(C)**; Protein expression of TNF-α and IL-6 after COS culture for 36 hours **(D)**. Determination of IC50 Value of Hydrogen Peroxide Damage to KGN Cells **(E)**. COS reduces H_2_O_2_-induced reactive oxygen generation in KGN cells **(F)**. COS inhibits the protein expression of HIF-1α and VEGFA in KGN cells in a dose-dependent manner **(G)**. *P<0.05, **P<0.01, ***P<0.001 *vs* Control group; ^#^ *P*<0.05, ^##^ *P*<0.01 *vs* Control group; ^#^ *P*<0.05, ^##^ *P*<0.01 *vs* H_2_O_2_ group.

We treated KGN cells with COS to evaluate the effect of COS on inflammatory and oxidative stress. The experiment of cell culture medium containing COS was divided into four groups. The Control group was the KGN cell culture group alone, 100 μg/mL group, 200 μg/mL group and 300 μg/ml group were the KGN cells cultured in COS medium containing 100 μg/mL, 200μg/mL and 300μg/mL concentrations in the medium. After being cultured at 24 hours and 36 hours, proteins were extracted to detect the protein expression of inflammatory factors. We found that COS promoted the expression of anti-inflammatory cytokines TGF-β1 and IL-10 in KGN cells in a dose-aging relationship, and the most obvious effect was at 300 μg/mL for 36 hours (**Figures 3A, B**). Meanwhile, COS inhibited the expression of pro-inflammatory cytokines TNF-α and IL-6 in KGN cells in a dose-aging relationship. The most pronounced effect was observed at a concentration of 300 μg/mL for 36 hours (**Figures 3C, D**).

In order to explore the ameliorative effect of COS on GCs oxidative stress, we established an H_2_O_2_-induced GCs oxidative stress model to simulate the GCs oxidative stress environment of PCOS patients. In this study, KGN cells were induced by different concentrations of H_2_O_2_ (100, 200, 300, 400, 500, 600μmol/L). CCK-8 cell viability experiment showed that the survival rate of KGN cells decreased logarithmically with the increase of H_2_O_2_ concentration. Survival curves were drawn according to the survival rates of KGN cells with different concentrations of H_2_O_2_, and the IC50 value was 300μmol/L. The oxidative damage model of KGN cells was established using an IC50 concentration of H_2_O_2 =_ 300 μmol/L to start subsequent grouping experiments (**Figure 3E**). Based on the established oxidative stress injury model, the protein expression of HIF-1α and VEGFA in KGN cells was detected after 24 hours culture with COS (100 μg/mL, 200 μg/mL, 300 μg/mL). The results showed that the expression of HIF-1α and VEGFA in the injury model group was significantly increased compared with that in the Control group, and the expression of HIF-1α and VEGFA decreased in a concentration-dependent manner after the addition of COS (**Figure 3F**). Based on the established oxidative stress injury model, KGN cells were cultured with COS (100 μg/mL, 200 μg/mL, 300 μg/mL) for 24 hours, and then the ROS Assay Kit was used to detect the level of ROS in KGN cells. The results showed that the fluorescence intensity of the damage model group was significantly higher than that of the control group, and the fluorescence intensity decreased with the addition of COS, especially when the COS was 200 μg/mL and 300 μg/mL (**Figure 3G**). These results indicate that COS can reduce the production of reactive oxygen species induced by H_2_O_2_ in KGN cells.

### 3.5 COS promotes KGN cells proliferation and inhibits apoptosis

**Figure 4:**
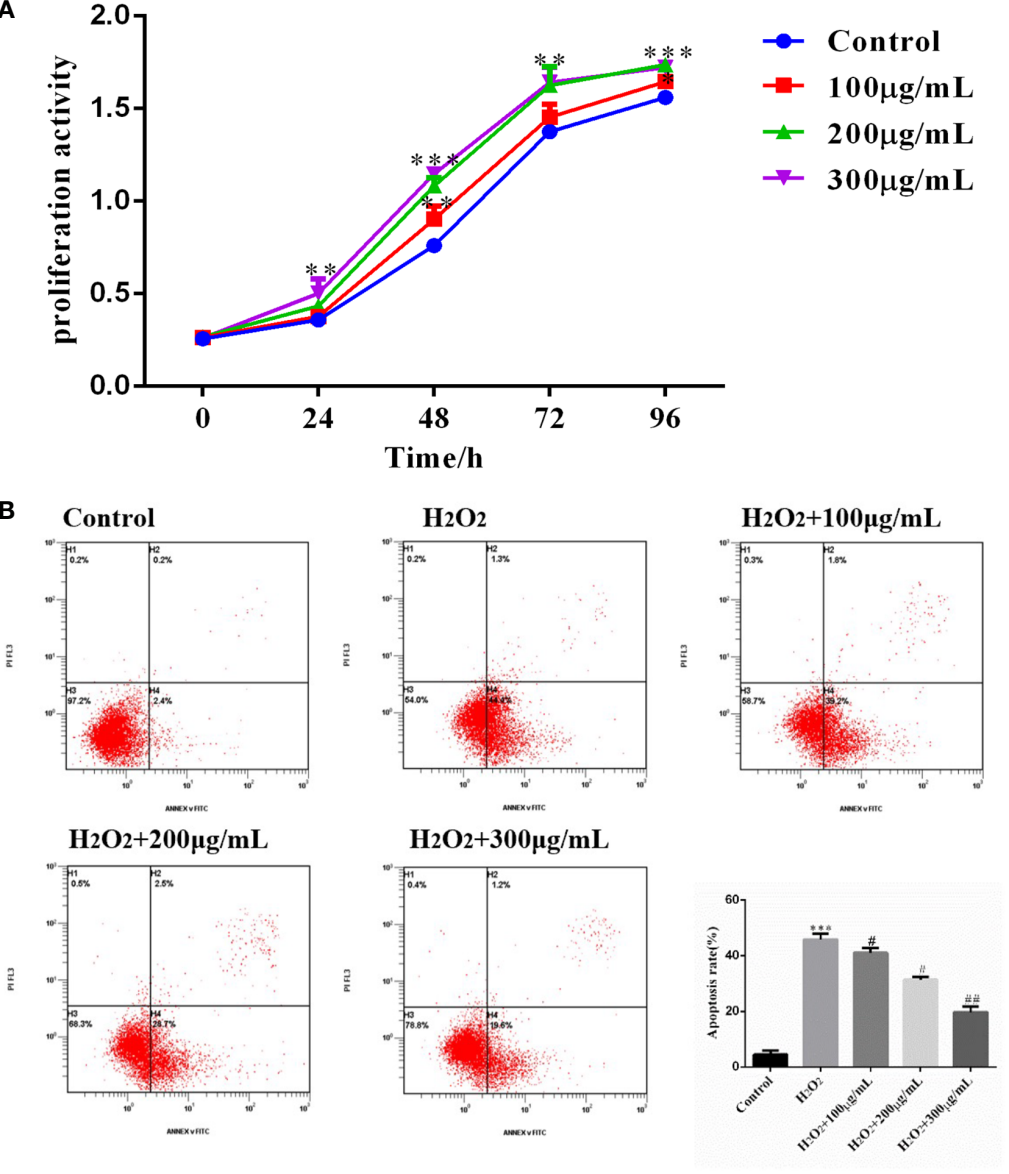
COS promoted KGN cell proliferation and inhibits apoptosis. The cell proliferation curve shows that the COS promotes the proliferation of KGN cells in a dose and time-dependent relationship **(A)**. COS reverse H_2_O_2_-induced apoptosis of KGN cells **(B)**. *P <0.05, **P <0.01, ***P <0.001 *vs* Control group; ^#^ *P*<0.05, ^##^ *P*<0.01 *vs* H_2_O_2_ group.

Cells from the above four groups were subjected to cell proliferation assay with OD at 450 λ, and analysis of variance by repeated measurements showed that cell proliferation was influenced by treatment duration and COS concentration (**Table 3**). Compared with the Control group, COS (100 μg/mL, 200 μg/mL, 300 μg/mL) promoted the proliferation of GCs in a dose-dependent manner. The OD values of 200 μg/mL and 300 μg/mL groups were significantly different from those of Control and 100μg/mL groups at 48 hours points (**Figure 4A**). On the basis of the established model of oxidative stress injury, KGN cells were cultured with COS (100 μg/mL, 200 μg/mL, 300 μg/mL) for 24 hours. The results showed that H_2_O_2_ significantly increased the apoptosis rate of KGN cells, 200 μg/mL and 300 μg/mL COS significantly reduced the apoptosis rate of KGN cells. This suggests that COS can reverse cell damage caused by oxidative stress (**Figure 4B**).

**Table 3:** CCK8 detected that chitosan oligosaccharides showed a dose and time-effect relationship to promote the proliferation of KGN cells.

Time	Control	100 μg/mL	200 μg/mL	300 μg/mL
0h	0.257 ± 0.005	0.264 ± 0.006	0.268 ± 0.11	0.261 ± 0.007
24h	0.359 ± 0.021	0.376 ± 0.010	0.434 ± 0.027	0.503 ± 0.064**
48h	0.760 ± 0.03	0.903 ± 0.057**	1.083 ± 0.036***	1.147 ± 0.023***
72h	1.377 ± 0.064	1.454 ± 0.057	1.626 ± 0.083**	1.643 ± 0.064**
96h	1.560 ± 0.044	1.647 ± 0.019*	1.738 ± 0.019***	1.723 ± 0.016***

*P <0.05, **P <0.01, ***P <0.001 vs Control group.

### 3.6 COS improves ovarian GCs inflammation and oxidative stress in PCOS patients

**Figure 5:**
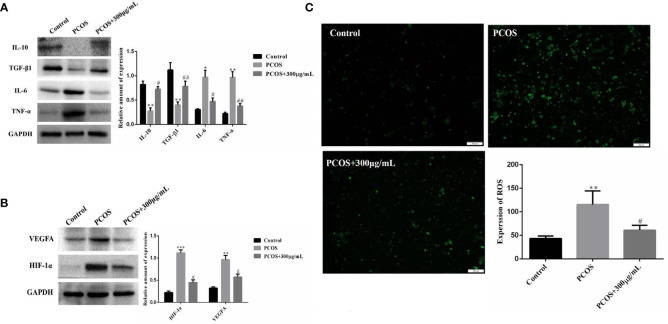
COS improves ovarian GCs inflammation and oxidative stress in PCOS patients. Effect of COS on the expression of inflammatory factors in ovarian GCs in patients with PCOS **(A)**. COS inhibits the expression of oxidative stress related factors in ovarian GCs of patients with PCOS **(B)**. COS decreases reactive oxygen species in GCs of patients with PCOS **(C)**. *P<0.05, **P<0.01, ***P<0.001 vs Control group; ^#^ *P*<0.05, ^##^ *P*<0.01 vs PCOS+300 μg/mL group.

The optimal COS concentration (300 μg/mL) and optimal culture time (36 hours) were used to culture the extracted GCs. We found that COS promoted the expression of anti-inflammatory factors TGF-β1 and IL-10, and inhibited the expression of pro-inflammatory factors TNFα and IL-6 in GCs of PCOS patients (**Figure 5A**). Meanwhile, the expression of oxidative stress-related factors HIF-1α and VEGFA was also decreased in GCs of PCOS patients (**Figure 5B**). Using ROS Assay Kit to detect their reactive oxygen levels, the fluorescence intensity of GCs in PCOS patients was significantly higher than that in control patients, while the fluorescence intensity of GCs in PCOS patients decreased after the addition of COS (**Figure 5C**), indicating that COS can reduce the ROS level of GCs in PCOS patients.

## 4 Discussion

**Figure 6:**
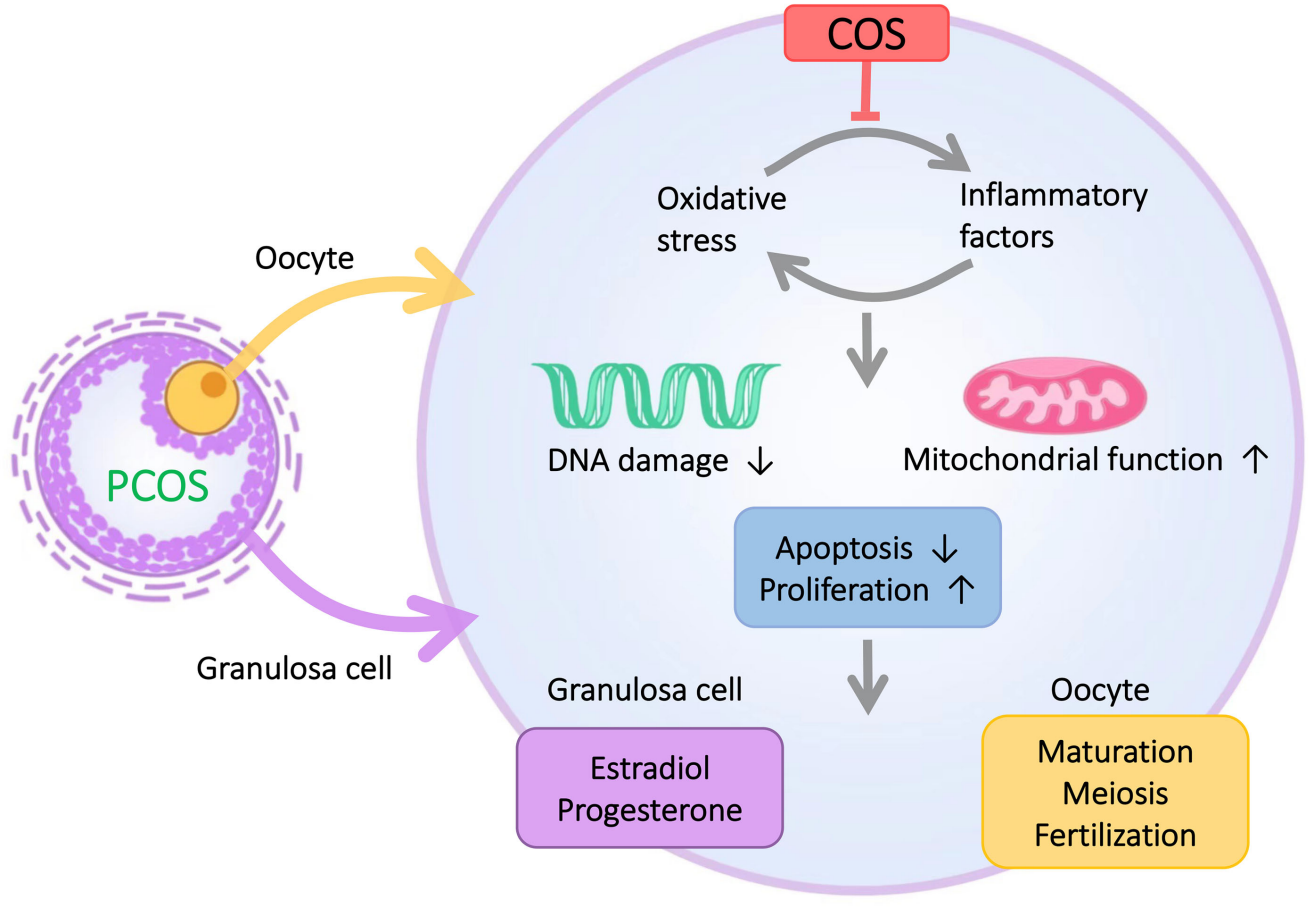
The microenvironment of GCs in PCOS patients is characterized by inflammation and oxidative stress. COS improves the inflammation and oxidative stress of GCs and thus enhances the proliferative capacity of GCs, reduces the apoptotic level of GCs, and ultimately improves abnormal follicular development in PCOS patients.

Polycystic ovary syndrome (PCOS) is the most common reproductive endocrine and metabolic disorder leading to infertility in women of reproductive age and is also one of the leading causes of anovulatory infertility. In this study, after analyzing clinical data collected from PCOS and control groups, we found that although the number of ovarian antrum follicles and a total number of eggs obtained were higher in PCOS patients than in the normal group, the cleavage rate was significantly lower than that in the normal group. This result suggests that there is abnormal follicular development in PCOS patients. However, the pathological causes are not fully understood. It is well known that GCs are essential for oocyte development, and GCs delivers amino acids and other nutrients to oocytes through gap junctions (24), which play a key role in important biological events such as oocyte meiosis, oocyte fertilization, and later embryonic dev elopement. Several studies have shown that the dysfunction of GCs will directly lead to abnormal follicular development in women with PCOS (3). For example, apoptosis of GCs not only promotes follicular atresia (3), but also leads to the generation of low-quality oocytes (25, 26). Previous studies have confirmed that chronic low-degree inflammation and oxidative stress are closely associated with the occurrence and development of abnormal PCOS follicles (27), and inflammation and oxidative stress can lead to increased apoptosis of GCs. In addition, oocyte was closely surrounded by GCs, implying important role of GCs in oocyte development. The increased apoptosis of GCs was closely related to the abnormal follicular development of PCOS. We hypothesize that the effects of inflammation and oxidative stress on follicular development in PCOS are likely to be mediated through the action of GCs. Therefore, to investigate in depth the causes of inflammation and oxidative stress leading to abnormal follicular development in PCOS, we focused on the relationship between GCs and inflammation and oxidative stress (**Figure 6**).

We found abnormal expression of inflammatory factors and oxidative stress-related factors in GCs of PCOS patients through the results of GCs extraction, and we found that the proliferation ability of GCs in PCOS group was weakened and the apoptosis level was increased. We found that the expression of TGF-β1 protein level was significantly lower than that of the control group, but the expression of mRNA was higher than that of the normal group. We hypothesized that the inconsistent expression of TGF-β1 gene and protein may be a consequence of the effect of ovulation induction drugs on GCs, or the inhibition of the translation of anti-inflammatory factor mRNA into protein in PCOS patients. The exact mechanisms involved need to be further explored. As key pro-inflammatory factors, abnormally elevated levels of TNF-α and IL-6 have been shown to inhibit the proliferation, differentiation, and maturation of PCOS follicles (28, 29). It has been reported that IL10 and TGF-β had anti-inflammatory properties and played a key role in disease prevention and autoimmunity (30, 31). Reduced expression of these two factors can result in inflammatory response, which leads to abnormal steroid synthesis and delayed follicular maturation and ovarian dysfunction. Conversely, TNF-α and IL-6, which belongs to pro-inflammatory factors, have negatively effects on follicular maturation, fertilization, embryonic implantation (29, 32). Increased expression of TNF-α induces apoptosis of antral follicular GCs, which leads to increased follicular membrane thickness and decreased granular layer thickness (32). In addition, IL-6 can reduce the activity of aromatase in the follicle, resulting in decreased concentration of estradiol in the follicle, fertility and fertilization ability (29). In our study, we find a significantly lower expression of IL10 and TGF-β and a obviously higher expression of TNF-α and IL-6 in PCOS patients, which suggests that one of the reason causing the abnormal biological functions of GCs in PCOS patients are probably associated with these abnormal inflammatory related factors. Hypoxia-inducible factor-1α (HIF-1α) is a functional subunit of hypoxia-inducible factor-1 (HIF-1) that degrades rapidly under normoxic pressure. Increased ROS levels can inhibit HIF-1α ubiquitination by inhibiting the activity of the proline hydroxylase family (PHDs), thus increasing HIF-1α levels *in vivo* (33). During follicular growth and development, low oxygen partial pressure in the follicular microenvironment can stimulate the expression of HIF-1α in ovarian GCs (34), which indicated an elevated ROS levels in GCs of PCOS patients. It was shown that HIF-1α plays a key role in follicular development and ovulation in mammals. HIF-1α is specifically expressed in ovarian cells and mainly in GCs, suggesting that it may be directly involved in the regulation of mammalian ovarian physiological function. In addition, this study also found that elevated gene expression of HIF-1α was detected in the ovaries of PCOS rats (35, 36). HIF-1α is also a central regulator of hypoxia stress response, and this transcription factor primarily regulates hypoxia-induced genes, including vascular endothelial growth factor A (VEGFA) (the most dominant and important member of VEGF) used in the angiogenic response (37). In the ovary, VEGF is mainly expressed in GCs and follicular membrane cells, but rarely expressed in mesenchymal cells (38). VEGF production is usually present in hypoxic cells and hypoxia is one of the most potent triggers of VEGF expression, involved in the transcription, stabilization, translation and release of VEGF (39). VEGF is a factor involved in normal reproductive function and follicular development, and it has been proved that VEGF levels in serum of PCOS patients are significantly increased (40, 41). As mentioned previously, increased expression of TNF-α in PCOS patients induces apoptosis in sinus follicle GCs (42), and the increase of oxidative stress can also lead to the increase of the apoptosis level of GCs. From this, we inferred that the abnormal follicular development in PCOS patients is likely due to inflammation and oxidative stress affecting the proliferation and apoptosis of GCs. In conclusion, we found that inflammation and oxidative stress exist in the microenvironment of GCs in PCOS patients, and the resulting decreased proliferation ability or increased apoptosis level of GCs is likely to be one of the important reasons for abnormal follicular development of PCOS. Thus, improving inflammation and oxidative stress in GCs is crucial for follicular development of PCOS patients.

KGN cells were cultured with different COS concentrations to evaluate the effect of COS on cells inflammation. We found that COS improved the proliferation and secretory function of cells in a dose-dependent manner, and this effect was most obvious at a COS concentration of 300 μg/mL for 36 hours. We assessed the ameliorative effect of COS on oxidative stress in cells by pretreatment with different concentrations of COS based on oxidative stress modeling. We found that COS increased the antioxidant and anti-apoptotic activity of cells in a dose-dependent manner. At the same time, our study showed that the COS group at 300 μg/mL still did not recover the level of the control group, indicating that COS did not completely reverse the damage of oxidative stress on cells and the reasons for this need to be further studied. It was found that COS could promote the expression of anti-inflammatory factors TGF-β1 and IL-10 in ovarian GCs of PCOS patients by culturing GCs from PCOS patients and control patients at the optimal concentration and time, Inhibited the expression of pro-inflammatory factors TNFα, IL-6, oxidative stress related factors HIF-1α and VEGFA, and decreased the level of reactive oxygen species.

## 5 Conclusion

In summary, we found inflammation and oxidative stress in the microenvironment of GCs from PCOS patients and found that GCs from PCOS patients had diminished proliferative capacity and increased levels of apoptosis. By using COS *in vitro* cell culture experiments, we found that COS can increase the expression of anti-inflammatory factors TGF-β1 and IL-10 and decrease the expression of pro-inflammatory factors TNFα and IL-6, as well as promote the proliferation of GCs. COS can reduce the level of reactive oxygen species in GCs under oxidative stress by inhibiting the expression of HIF-1α and VEGFA and suppressing the apoptosis of GCs induced by oxidative stress. Finding COS new pharmacological application in infertility treatment is expected to provide a new therapeutic treatment for clinical PCOS patients by enhancing the GCs proliferative capacity and decreasing their apoptosis level *via* improving the inflammation and oxidative stress of GCs into a suitable intensity.

## Data availability statement

The original contributions presented in the study are included in the article/supplementary material, further inquiries can be directed to the corresponding author/s.

## Ethics statement

The studies involving human participants were reviewed and approved by Medical Ethics Committee of Jiangxi Maternal and Child Health Hospital. The patients/participants provided their written informed consent to participate in this study.

## Author contributions

YZ, JT, and ZSZ developed the concept and design. QX and WH performed most of the experiments and wrote the manuscript draft. YL participated in the manuscript preparation and editing. SL, ZQZ, YD, WW, and RW performed some of the experiments and provided critical discussion of the manuscript. All authors contributed to the article and approved the submitted version.
